# Dual-modality SEM-Raman smart scanning for fast hyperspectral Raman micro-imaging – application to bones

**DOI:** 10.1364/BOE.551298

**Published:** 2025-02-07

**Authors:** Valentin Gilet, Guillaume Mabilleau, Matthieu Loumaigne, Raffaele Vitale, Thomas Oberlin, José Henrique de Morais Goulart, Nicolas Dobigeon, Cyril Ruckebusch, David Rousseau

**Affiliations:** 1 Université d’Angers, LARIS, UMR IRHS INRAe, 49000 Angers, France; 2 Université d’Angers, Nantes Université, Oniris, Inserm, RMeS, REGOS, SFR ICAT, F-49000 Angers, France & CHU Angers, Bone Pathology Unit, F-49933 Angers, France; 3 Université d’Angers, MOLTECH-Anjou, UMR CNRS 6200, 49000 Angers, France; 4 Univ. Lille, CNRS, LASIRE, F-59000 Lille, France; 5 Université de Toulouse, ISAE-SUPAERO, 31400 Toulouse, France; 6 Université de Toulouse, IRIT/INP-ENSEEIHT, 31071 Toulouse, France

## Abstract

Recent works on smart scanning techniques in Raman micro-imaging demonstrate the possibility of highly reducing acquisition time. In particular, Gilet *et al.* [Optics Express 32, 932 (2024)10.1364/OE.50973638175114 ] proposed a protocol combining compression in both spectral and spatial domains by focusing on essential information. This protocol consists of a two-pass scan in Raman modality at different signal-to-noise ratios (SNR). The first scan of the entire sample area at low SNR, and was identified as the bottleneck of the whole process. We propose revisiting this protocol by replacing this first scan with scanning electron microscopy (SEM), which is a faster imaging modality. We demonstrate that acquiring real data of biomedical interest according to this new protocol is three times faster, with limited distortion on the reconstructed Raman spectra and preserved clinical value of the extracted information. This is illustrated on bone samples for which SEM is correlated with Raman. We discuss the potential extension of this method to other slow spectral imaging modalities conventionally based on raster scans.

## 1. Introduction

Despite providing interesting chemical information in a contact-less and non-destructive way, hyperspectral Raman micro-imaging suffers from very long acquisition times due to its point-by-point raster scanning [1]. Indeed Raman scattering is known to be a rare phenomenon, with only a proportion of $10^{-7}$ of the photons being scattered. A current open problem of science lays in the acceleration of this acquisition [2] which, by extension, tends also to reduce photo-damage induced by the light onto the sample [3].

Some authors manage to speed up the acquisition process by enhancing Raman scattering via resonance of the signal, e.g. with coherent Raman scattering [4,5] or with surface plasmon resonance [6,7]. Others enhance the signal by reducing the temperature of the targeted samples [8]. A gain in speed can also be obtained via parellelization, with optical devices producing line-illumination [8,9] or array of focused laser beams [10]. More, one can mention the use of frequency domain by sending probe pulses onto the sample [11] or also the use of full-field coherent illumination [12–14]. Alternatively, some acceleration can be brought numerically, e.g. as post-processing of fast acquisition at low signal-to-noise ratio (SNR) via deep learning [15–17], or with the use of matrix decomposition and matrix completion [18].

Yet another approach lays in the smart scanning computational approach as developed recently in [19–22]. In these works, the authors demonstrate the possibility of accelerating the acquisition process by only looking for some specific information in a sample. More precisely, it has been shown that only a few spectral pixels (called essential spectra pixels) are required to reproduce all measured spectra by convex linear combination [19,20].

The selection of these essential spectra pixels was shown accessible on-the-fly [21] via a computation in the Fourier phasor domain. A first fast scan of the whole sample is performed to identify, after computation, the so-called essential pixels. A second longer scan, is then performed only on these essential pixels. These high-quality spectra are used to reconstruct the spectra of the rest of the pixels. In [22] this approach was implemented on simulated and real samples with an estimated 100-fold reduction of acquisition time. In [22], the bottleneck, in terms of time, of this smart scanning approach was identified as the first scan of the whole sample. As suggested in [22], it should be possible to further speed up the scanning by replacing the first scan by another fast imaging modality. This work investigates this path with the use of scanning electron microscopy (SEM) coupled with Raman and applied to bone samples for which the Raman classical raster scan is known to be very slow. The article is structured in the following way: Section 2 presents the acquisition protocol and the real-world samples of biomedical importance used to illustrate the interest of the protocol. Section 3 demonstrates the gain of speed while ensuring a high-quality Raman spectrum reconstruction and clinical value. In addition to this proof of feasibility, a possible extension of this dual modality smart scanning protocol is discussed in Section 4. Section 5 concludes the paper.

## 2. Material and methods

### 2.1. Three-pass acquisition protocol

**Fig. 1.**
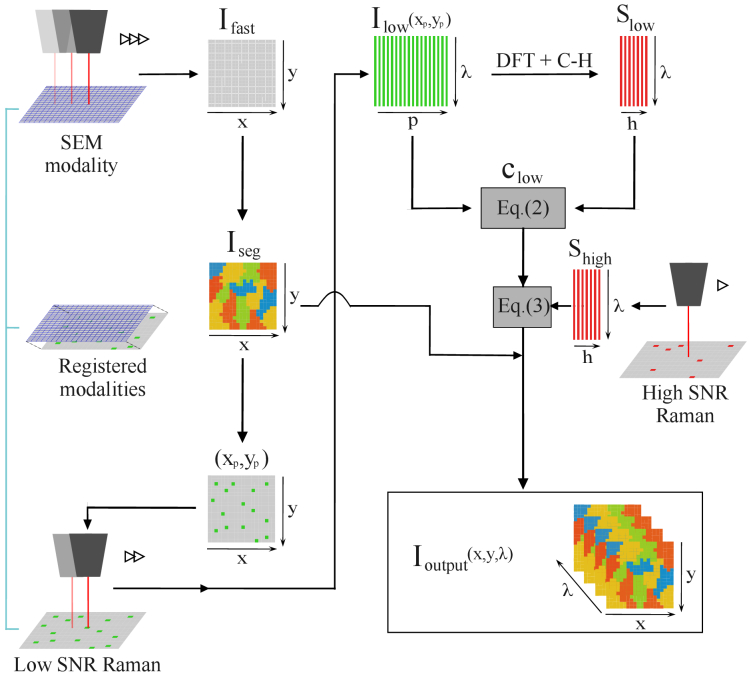
Our proposed Three-Pass protocol. First, the sample is acquired in a modality which allows a fast acquisition (here chosen as SEM). The obtained image is then segmented into superpixels to select superpixels centroids positions. Selected positions are quickly acquired in Raman modality leading to ${\text{I}}_{\text{low}}$ , the matrix of low SNR spectra comprising P spectra indexed by p . The search of essential spectra is made on ${\text{I}}_{\text{low}}$ leading to ${\text{S}}_{\text{low}}$ , the matrix of low SNR essential spectra comprising H spectra indexed by h . ${\text{I}}_{\text{low}}$ are unmixed with respect to ${\text{S}}_{\text{low}}$ to obtain ${\text{c}}_{\text{low}}$ , the matrix of concentrations in essential spectra of each spectrum. A second acquisition in Raman, longer than the first one, is performed on the positions of essential spectra leading to ${\text{S}}_{\text{high}}$ , the matrix of high SNR essential spectra. Finally, ${\text{I}}_{\text{output}}(x,y,\lambda )$ is produced by the combination of the matrix of superpixels centroids spectra obtained with Eq. (3) and the spatial location of each superpixels carried in ${\text{I}}_{\text{seg}}$ .

We propose the acquisition protocol depicted in Fig. 1. First, an imaging modality, distinct from Raman, is used to produce a first image denoted ${\text{I}}_{\text{fast}}$ . In this article the fast modality is chosen as the SEM imaging. The acquired image ${\text{I}}_{\text{fast}}$ is then segmented into P superpixels, i.e. blocks of neighboring pixels labelled from 1 to P , to produce ${\text{I}}_{\text{seg}}$ . Similarly to the approach followed in [22], for each superpixel a representative pixel is selected among all pixels constituting a superpixel. In our case the centroid of each superpixel is chosen as its representative. The position $\left(x_{p},y_{p}\right)$ , with p ranging from 1 to P , of each representative pixel represents a subset of the whole matrix of pixels. We assume that this subset is a good representative of the spectral diversity of the sample. This means that the contrast of the fast imaging modality (SEM here) should by correlated with the contrast obtained in Raman microscopy. We also assume that the fast imaging modality is registered with the Raman microscopy scanning referential. Another assumption, which is worth mentioning, is that like for any multi-pass acquisition protocol, the sample is supposed to be fixed and the micro-stage positioning accurate enough so that it is possible indeed to scan several times the same pixels at different SNR. Based on these assumptions, Raman spectra are acquired at each representative pixel position $\left(x_{p},y_{p}\right)$ at low SNR to create a matrix ${\text{I}}_{\text{low}}(x_{p},y_{p})$ with P spectra along the columns made of L spectral bands along the rows. As proposed in [22], a discrete Fourier transform (DFT) is then applied to ${\text{I}}_{\text{low}}(x_{p},y_{p})$ along the spectral axis. It reads

(1) $$I_{\text{low}}{\left(x_{p},y_{p}\right)}_{\lambda }=\frac{1}{L}\sum_{r=1}^{L}(G_{r}(x_{p},y_{p})+iQ_{r}(x_{p},y_{p}))\exp\left(i\frac{2\pi }{L}(\lambda -1)(r-1)\right)\;,$$

with i the unit imaginary number and $G_{r}(x_{p},y_{p})$ and $Q_{r}(x_{p},y_{p})$ the real and imaginary parts of the Fourier coefficients of the pixel located at $\left(x_{p},y_{p}\right)$ of the r -th harmonic. For a given harmonic indexed by r , the point cloud depicted by $G_{r}(x_{p},y_{p})$ and $Q_{r}(x_{p},y_{p})$ , normalized following L1-norm, allows discriminating the different chemical contents constituting the sample. The spectra corresponding to the points laying on the convex-hull (C-H) of the points cloud represent the so-called essential spectra [21]. They constitute another subset of pixels gathered in ${\text{S}}_{\text{low}}$ , a matrix of H spectra along the columns made of L spectral bands along the rows.

The subset of essential spectra ${\text{S}}_{\text{low}}$ contains essentially a smaller number of spectra than the subset of ${\text{I}}_{\text{low}}$ . Hence, one is able to obtain $c_{\text{low}}$ , a matrix containing H concentrations in essential spectra ${\text{S}}_{\text{low}}$ along the rows of the P spectra of ${\text{I}}_{\text{low}}(x_{p},y_{p})$ , by unmixing methods. As in [22] we used a simple least-squares estimator expressed as

(2) $$c_{\text{low}}(x_{p},y_{p})={\left({\text{S}}_{\text{low}}{\text{S}}_{\text{low}}^{T}\right)}^{-1}{\text{S}}_{\text{low}}{\text{I}}_{\text{low}}\left(x_{p},y_{p}\right).$$

The last step of the proposed acquisition protocol consists in the rescan of the H essential spectra during a longer exposure to obtain a higher SNR. This produces ${\text{S}}_{\text{high}}$ a matrix containing a high-SNR acquisition of the same spectra as ${\text{S}}_{\text{low}}$ , allowing to reconstruct ${\text{I}}_{\text{output}}(x,y,\lambda )$ an hyperspectral image (HSI) made of a stack of L times ${\text{I}}_{\text{seg}}$ for which each pixel of the superpixel p corresponds to the high-SNR spectrum

(3) $${\text{I}}_{\text{output}}(x_{p},y_{p})={\text{S}}_{\text{high}}^{T}c_{\text{low}}(x_{p},y_{p})\;,$$

for 1,…,P .

The acquisition protocol of Fig. 1 should be compared with various existing scanning techniques. The first scanning technique is the standard current approach in raster scanning with Raman hyperspectral microscopy which trivially scans the sample at high SNR for slow but accurate hyperspectral imaging or at low SNR for fast but noisy hyperspectral imaging. A second scanning technique is the smart scanning approach recently proposed in [22]. This approach follows the proposed protocol, but with ${\text{I}}_{\text{fast}}$ corresponding to the full raster scan of the image at low-SNR instead of using another imaging modality. This approach is therefore a Two-Pass only acquisition protocol where the bottleneck in terms of scanning time is the first pass at low SNR. We expect the protocol of Fig. 1 to be faster than the high SNR scan due to the implementation of spatial compression (selection of superpixels) and spectral compression (selection of essential spectra in the Fourier domain). Compared to the Two-Pass scan, the new method is expected to be faster primarily because of the use of a fast imaging modality, which significantly reduces acquisition time relative to a full raster scan, even at low SNR.

### 2.2. Practical use-case

The smart scanning protocol proposed in the previous section is appealing in principle but clearly requires a practical use-case to demonstrate its efficiency in the real world. To be relevant, this practical use-case must involve samples for which the Raman acquisition is indeed very long so that the expected gain in acquisition time would be of practical interest. We selected bones samples collected after total hip replacement arthroplasty with written informed consent for routine clinical pathology. Bone samples were fixed with 4% paraformaldehyde for 24h and embedded undecalcified in polymethylmethacrylate resin. Bone blocks were transversely cut with a diamond saw, grinded with SiC papers of ascending grades and polished with diamond paste. After a rapid cleaning with milliQ water, the bone sample is ready for imaging [23].

**Fig. 2.**
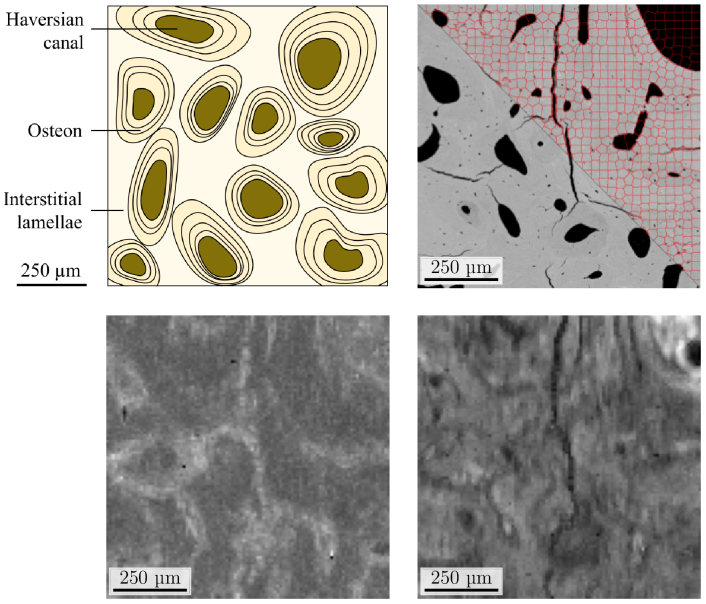
Anatomical board of a cortical bone sample (upper-left). Acquisition of the same sample in, SEM modality (upper-right) and Raman modality at low SNR (bottom-left) and high SNR (bottom-right). For visualization purposes, the hyperspectral image obtained during the Raman acquisition is averaged along the spectral axis and SEM image is shown at its full resolution. An example of a superpixel segmentation is shown on the upper-right part of the SEM image.

This type of samples, as demonstrated in [22], are known to require very long acquisition at high SNR. For instance, in cortical bone, cylindrical structure, known as osteon ( ≈ 0.10-0.15 ${\mathrm{mm}}^{2}$ ), corresponding to active bone areas (Fig. 2) requires at least 14 hours of acquisition with the current raster scan for an analyzable image [24]. On the other hand, bones can be imaged with various imaging techniques faster than Raman microscopy: this includes microcomputed tomography, SEM, optical microscopy, including confocal laser scanning microscopy and Fourier transform infrared microspectroscopy with a focal plane array detector. As described in Figure 1, we decided to use SEM images as a first fast modality since it was recently shown in [25] that the degree of tissue mineralization, measured as the content of calcium in the bone matrix is highly correlated to ratio of Raman peaks of interest for clinical diagnosis. Moreover, it should be noticed that registered SEM - Raman microscopies now starts to be available in commercial setups, such as the Renishaw ${\mathrm{inLux}}^{\text{TM}}$ which allows to switch from one modality to another in half a minute [26]. It is therefore possible to envision an acquisition of the exact same scene of a sample in both Raman and SEM modalities as described in the acquisition protocol of Fig. 1. However, in this article, for a first proof of feasibility, the proposed Three-Pass protocol of Fig. 1 was simulated based on the real SEM and Raman images acquired on the real bones samples described in this section.

### 2.3. Data set

To assess the acquisition protocol described in the previous section 2.1, we built a data set composed of 12 SEM images associated with Raman HSI at low and high SNR, from which we obtain ${\text{I}}_{\text{fast}}$ , ${\text{I}}_{\text{low}}$ and thus ${\text{S}}_{\text{low}}$ , and ${\text{S}}_{\text{high}}$ . As presented on Table 1, images have been acquired on six different samples. Three samples were retrieved from patients suffering of type-2 diabetes mellitus, known to present higher tissue mineralization. The three remaining bone samples were collected from patients suffering of osteoarthritis with normal femoral neck bone. Bone blocks were surface-polished as described in [25]. SEM images were acquired with an accelerating voltage of 20 keV, a current probe of 250 pA measured at the sample surface and a magnification of 50× in high vacuum mode with a Zeiss EVO LS10 microscope. Originally, each of these images captures a very large area of each sample ( $\approx \;15{\text{mm}}^{2}$ ). Raman HSI were then acquired with a Renishaw InVia Qontor confocal Raman microscope, using a 20× Olympus objective (0.40 N.A.), a spectrometer equipped with a 1800 g/mm grating and a 785 nm laser at 3 mW. As shown in Table 1, two small areas ( $\approx \;1{\text{mm}}^{2}$ ) were acquired on each sample. Each area have been acquired at low SNR, i.e. 100 ms per point, and high SNR, i.e. 5000 ms per point. Quantitatively, SNR is defined as follows:

(4) $$\text{SNR}=\frac{1}{P}\sum_{p=1}^{P}10\;{\text{log}}_{10}\left(\frac{\sum_{\lambda =1}^{L}I_{\text{S}\lambda }{\left(x_{p},y_{p}\right)}^{2}}{\sum_{\lambda =1}^{L}e_{\lambda }{\left(x_{p},y_{p}\right)}^{2}}\right)$$

with ${\text{I}}_{\text{S}}$ the signal extracted from ${\text{I}}_{\text{low}}$ by the Savitzky-Golay algorithm [27], and e the noise estimated by the difference of ${\text{I}}_{\text{low}}$ and ${\text{I}}_{\text{S}}$ . The SNR of an HSI is estimated by the average SNR of the whole spectra comprised in the HSI. Thus, average over the 12 images, low SNR and high SNR respectively give SNR values of 8.7 and 23.3. SEM and Raman images were then manually registered and cropped to about 400×400 pixels to match the Raman acquisition area, and then resized at 100×100 pixels to match the images’ spatial dimension. Raman hyperspectral images contain also a spectral dimension of 940 pixels. An example of a bone sample acquired in SEM and Raman microscopy at low and high SNR is provided in Fig. 2 for illustration. The Raman HSI is averaged in the spectral dimension to produce a single component image. Even with this very crude representation, the correlation between the spatial contrast in the SEM and Raman imaging modalities is clearly visible (as quantified in [25]).

**Table 1.** Details about the data set used in this work.

			**Images dimensions**			
	Sample	Area	Original SEM image	Registered SEM image	Raman Low SNR HSI	Raman High SNR HSI
Diabetic	$D_{1}$	Z1	1040×1836	421×421	100×100×940	100×100×940
		Z2		422×422		
	$D_{2}$	Z1	2055×724	383×383		
		Z2		444×444		
	$D_{3}$	Z1	1027×1510	448×448		
		Z2		444×445		
Healthy	$D_{4}$	Z1	2048×1536	442×442		
		Z2		450×447		
	$D_{5}$	Z1	2048×1536	465×466		
		Z2		468×468		
	$D_{6}$	Z1	2048×1536	439×427		
		Z2		448×448		

For this data set, hyper-parameters of the different algorithms involved in the Two-Pass and Three-Pass protocols were defined as follows:

• for superpixelization, we used Simple Linear Iterative Clustering (SLIC) as provided in [28] with the following parameters: the number of superpixels is set such that obtaining superpixels of approximately 3×3 pixels ( $\approx \;30\times 30\;\mu {\text{m}}^{2}$ ) in order to preserve sufficient image definition; The size of the superpixels has to be chosen smaller than the structure of interest, i.e. a scale for which the chemical content can be assumed to be homogeneous. In the case of the bone samples this is the bone lamellae. The compactness value is set as 0.1 to give more importance to grey level during the segmentation and avoid squared superpixels; a sigma value is fixed to 0 to avoid smoothing the image before the segmentation and missing important information.
• for the search of essential information, we only exploited the first harmonic, i.e. r=2 in Eq. (1).

### 2.4. Evaluation metrics

As evaluation metrics of the quality of the reconstructed spectra, we decided first to assess the global reconstruction of the entire HSI with a simple root mean square error (RMSE)

(5) $$\text{RMSE}=\frac{1}{XY}\sum_{x=1}^{X}\sum_{y=1}^{Y}\sqrt{\frac{1}{L}\sum_{\lambda =1}^{L}{\left[{\text{I}}_{\text{output}}(x,y,\lambda )-{\text{I}}_{\text{high}}(x,y,\lambda )\right]}^{2}}$$

which measures globally the spectral error between reconstructed HSI ${\text{I}}_{\text{output}}$ and the spectral ground truth, i.e. HSI that would have been acquired on each pixel at high SNR, ${\text{I}}_{\text{high}}$ .

The relevance of the RMSE criterion is limited, since the global reconstruction of the spectra is not a guarantee of the clinical value of the information which may be located in some specific part of the spectra as in our use case. Therefore, in order to assess the clinical value of the results, as suggested in [25], we also decided to evaluate the restitution of a parameter essential to bone specialists: the ratio image

(6) $$R(x,y)=\frac{{\text{I}}_{\text{output}}(x,y,\lambda_{1})}{{\text{I}}_{\text{output}}(x,y,\lambda_{2})}$$

corresponding to the ratio of the $\nu_{1}{\text{PO}}_{4}$ spectral peak ( $\lambda_{1}=960$ ${\mathrm{cm}}^{-1}$ ) and ${\text{CH}}_{2}$ peak ( $\lambda_{2}=1450$ ${\mathrm{cm}}^{-1}$ ) intensities.

The proposed Three-pass scanning protocol assumes that the fast scan (SEM here) is correlated with the slow scanning modality (Raman here). We quantified this correlation with the following normalized cross-covariance coefficient

(7) $$\text{C}(A,B)=\frac{\text{Cov}(A,B)}{\sqrt{\text{Var}(A)\cdot \text{Var}(B)}},$$

between pairs of images A and B . The normalized cross-covariance coefficient culminates at 1 for maximally correlated images or −1 respectively for anti-correlated images and shrinks to zero for independent images. We measured this normalized cross-covariance coefficient between the SEM and the ratio images R(x,y) of Eq. (5) computed from the Raman microscopy and compared this value with the similarity between the high SNR and low SNR ratio R(x,y) (which corresponds to the image pair used in the Two-Pass protocol). As shown in Table 2 the normalized cross-covariance between SEM and High SNR ratio image R(x,y) reaches a relatively high value close to the self similarity of the ratio images R(x,y) computed in the low and high SNR regime. This further validates, as demonstrated in [25] with other samples, the relevant association of SEM and Raman for the illustrative use case chosen in this work.

**Table 2.** Similarity between pairs of images.

Images pair	C (A,B) of Eq. (8)^*a*^
SEM / High SNR R(x,y) ratio	0.60 ± 0.16
Low SNR R(x,y) ratio / High SNR R(x,y) ratio	0.73 ± 0.16

^*a*^ given values correspond to the mean ± standard deviation over the entire data set.

As demonstrated in [25], an histogram of the R(x,y) image can be calculated as

(8) $$\hat{f}(\nu )=\frac{1}{n\delta }\sum_{i}^{n}⊮\left(\left|\frac{\nu -\nu_{i}}{\delta }\right|\leq \frac{1}{2}\right)\;,$$

with ν represents the mean of each bin having a width of δ , n represents the number of pixels involved in the computation, and ⊮(⋅) represents the indicator function. $\hat{f}(\nu )$ gives an estimation of the tissue mineralization of the sample, that is highly correlated with the Calcium concentration, i.e. the tissue mineralization measured by the gray levels provided by the SEM. Based on reference bone samples, this parameter $\hat{f}(\nu )$ can be used to distinguish normal tissue from tissue hypermineralization in bone pathology.

Once the histogram in Eq. (8) is computed, on can assess how the obtained results can be used to distinguish between healthy and diabetic samples. To this end, we measured, for the different tested acquisition protocols, the Bhattacharyya distance between the distribution of the $\hat{f}(\nu )$ parameter for these two normalized probability distributions $\hat{f}(\nu \in \text{Healthy})$ and $\hat{f}(\nu \in \text{Diabetic})$ . The Bhattacharyya distance $D_{B}$ reads

(9) $$D_{B}=-\log\left(\sum_{\nu_{i}\in \nu }\sqrt{\hat{f}(\nu_{i}\in \text{Healthy})\hat{f}(\nu_{i}\in \text{Diabetic})}\right)\;,$$

and is a common metric to distinguish noisy data in optics specially when they are expected to be non-Gaussian [29]. The higher the $D_{B}$ , the more distinguishable are the diabetic samples from the healthy ones and, therefore, the more reliable the diagnosis based on the R ratio will be.

In addition to these metrics, we assess the processing time taken by the full acquisition scheme. This includes the acquisition time of the first pass via the SEM modality, the Raman scans at low and high SNR (including an estimation of the duration of micro-stage transfer), the computation time taken for the segmentation in superpixels and the essential information search.

All metrics discussed in this section, in addition to qualitative visualization of the spatial and spectral data, were computed for the proposed Three-Pass protocol and compared to the Two-Pass, to the Low-SNR scan, and to so-called Reference scan that would have been entirely acquired at high SNR.

## 3. Results

**Fig. 3.**
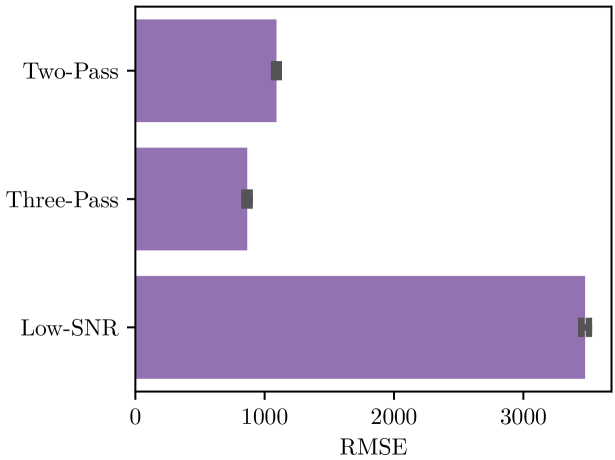
Evaluation of the spectral quality by comparison with the Reference acquisition via the RMSE for the Two-Pass protocol [22], the Low-SNR protocol and for the proposed Three-Pass protocol. RMSE has been calculated on the 12 samples constituting the data set, each bar height represents the average RMSE and the error bar represents a 95% confidence interval of the whole pixels for each method.

**Fig. 4.**
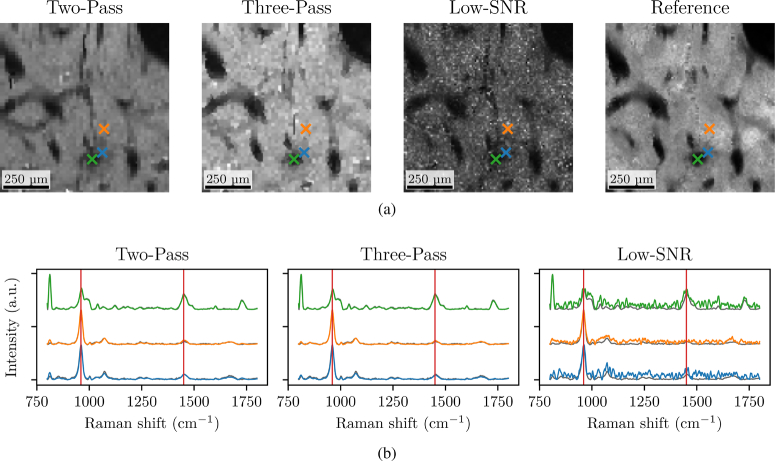
Qualitative assessment of the spatial reconstruction panel (a) and spectral reconstruction panel (b) for the different acquisition protocol. Images shown on (a) correspond to the R(x,y) ratio image of (3) obtained from the ratio of image at the 960 ${\mathrm{cm}}^{-1}$ and 1450 ${\mathrm{cm}}^{-1}$ spectral bands. These spectral values are marked as red vertical lines on panel (b). Spectra depicted on (b) correspond to the pixels marked with color matching cross on (a). The Reference spectra are provided in black on panel (b).

We start with the assessment of our smart acquisition protocol with the global reconstruction of the data measured with the RMSE of Eq. (5). The proposed Three-Pass method is compared with the Low-SNR acquisition and the Two-Pass acquisition protocol of [22]. The RMSE for these three acquisitions protocols are shown in Fig. 3. The Two-Pass and Three-Pass protocols demonstrate similar errors. This demonstrates that the correlation between the SEM and the Raman signals, while slightly lower than in the Two pass protocol (as shown in Table 2) still enables a relevant selection of pixels. As a complement to the quantitative measure of the global RMSE, Fig. 4 provides a visual assessment of the spatial and spectral quality of the reconstruction of the images and spectra for the different tested protocols.

**Fig. 5.**
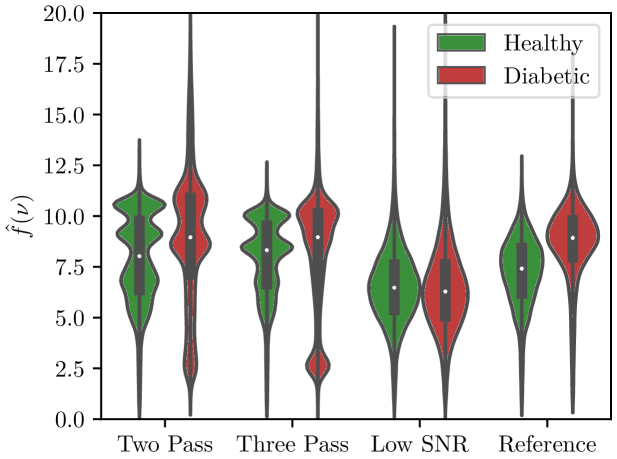
Distribution of the pixels of R of interstitial lamellae and osteon regions, taken over all the images data set, for each method. Unlike in (6), distributions are here smoothed by a Gaussian kernel [30]. Colors are assigned to differentiate pixels from healthy and diabetic samples. For each distribution, the white dot corresponds to the mean, the black rectangle corresponds to the standard deviation and the black line to the whisker.

We now move to the assessment of the spectral quality of the data on the specific $\hat{f}(\nu )$ parameter representing the tissue mineralization. Figure 5 shows the distributions of these ratio for the healthy and diabetic samples. The distributions are clearly distinct between healthy and diabetic in the Reference scanning protocol as demonstrated in [25]. Noise in the case of the Low-SNR protocol highly affects the distributions and hence makes it impossible to distinguish between healthy and diabetic samples. This distinction between healthy-diabetic is preserved in the Two-Pass and Three-Pass protocol. Table 3 gives, the Bhattacharyya distance $D_{B}$ , i.e. a quantitative value of the overlap between the distributions of $\hat{f}(\nu )$ for the healthy and diabetic samples, for each acquisition protocol. Interestingly, the Bhattacharyya distance $D_{B}$ appears to be higher for the Three-Pass and the Two-Pass protocols than the Reference one. The Three-Pass (as also the Two-Pass) acquisition protocol while showing a distortion on the RMSE by comparison with the Reference protocol preserves the clinical diagnosis value of the spectra.

**Table 3.** Bhattacharyya distance between healthy and diabetic samples for each method^*a*^

Method	$D_{B}$
Two-Pass	0.163
**Three-Pass**	**0.130**
Low-SNR	0.009
Reference	0.126

^*a*^ Higher is the value, lower is the overlap between distributions.

**Fig. 6.**
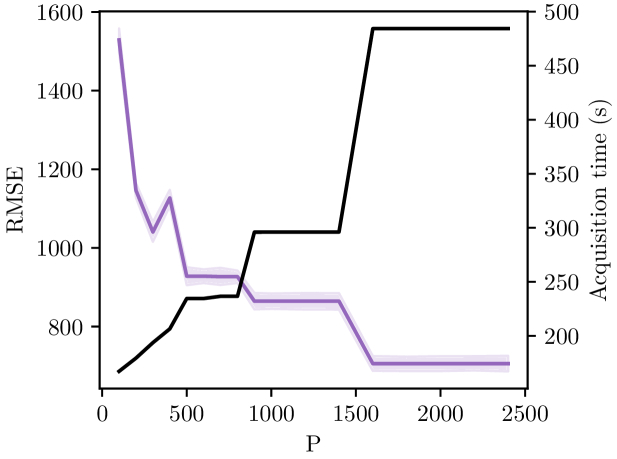
Evolution of the RMSE value, represented by the purple line, and the total acquisition time, represented by the black line, as a function of the number P of superpixels. RMSE has been calculated on the 12 samples constituting the data set, line plot represents the average RMSE and the transparent area represents a 95% confidence interval of the whole pixels for each method.

Last but not least, the acquisition time is provided in Table 4 and clearly demonstrates the superiority of our proposed Three-Pass scanning protocol. The gain brought by this protocol is a factor of nearly 100 in comparison with the standard current Reference protocol and a factor of almost 3 in comparison with the Two-Pass protocol of [22]. As a complement, Fig. 6 depicts the impact of the parameter P on the reconstruction and the acquisition time. An interesting trade-off between RMSE and acquisition time can be found around P=1000 , value that we set to have superpixels with a shape of approximately 3 by 3 pixels.

**Table 4.** Detailed time taken by each acquisition protocol to produce an image of bones with 100×100 pixels and 940 spectral bands.^*a*^,^*b*^

	First	LSNR	Proces.	HSNR	Total
	Modality	Pass	Time	Pass	
Two-Pass	0.0	1300.0	9.1	58.3	1367.4
**Three-Pass**	**120.0**	**335.9**	**19.6**	**49.6**	**525.1**
Low-SNR	0.0	1300.0	0.0	0.0	1300.0
Reference	0.0	0.0	0.0	50000.0	50000.0

^*a*^ First modality refers to the pass in SEM modality, LSNR and HSNR refer respectively to the low and high SNR pass, and Proces. Time corresponds to the time taken to process data.

^*b*^ Time is given in seconds.

## 4. Discussion

The previous section demonstrated, on bone samples, the added value of the proposed Dual-modality SEM-Raman smart scanning protocol for fast and yet accurate hyperspectral Raman microscopy in comparison with the current long raster scan and also with the recent smart scanning single modality protocol introduced in [22]. Let us now discuss the possible limitations of this scanning protocol and the perspectives of further developments.

**Fig. 7.**
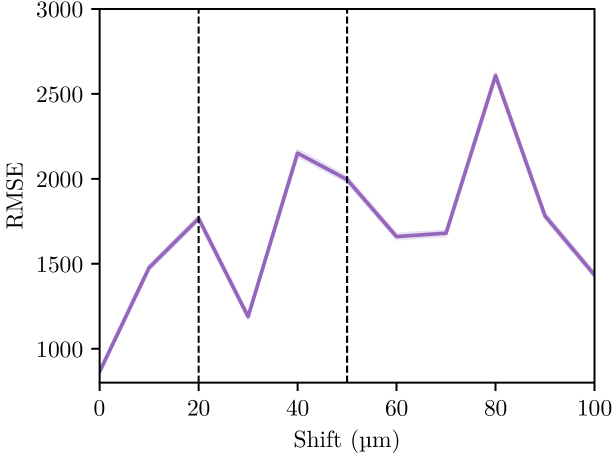
Evolution of the RMSE value according to a wrong registration of the SEM and Raman modalities. The error is simulated by a shift of n pixels (10 μ m per pixel) between the two modalities. Vertical black dashed lines represent approximately the size of a superpixel. RMSE has been calculated on the 12 samples constituting the data set, line plot represents the average RMSE and the transparent area represents a 95% confidence interval of the whole pixels for each method.

So far, we have considered that the two modalities (SEM and Raman) could be registered. This was manually done here but it is now available automatically in commercial setups. One could wonder about the impact of possible misalignment between the two modalities prior to the second and third passes. As shown in Fig. 7, the error tends to increase slightly as the degree of misalignment grows. In fact, the error evolution seem related to the size of a superpixel, which is approximately $30\times 30\;\mu {\text{m}}^{2}$ . The chemical content of the bone samples turns out to be relatively stationary at this chosen size of superpixel. It is also important to mention, as a possible limitation of any multiple pass algorithm that some spectral change could occur with autofluorescence, photobleaching or resonance scattering. These aspects did not affected the clinical value of the measured spectra as demonstrated in Fig. 5 and Table 3.

For this proof of concept of our smart scanning protocol, acquisitions were simulated from real images. The estimated time takes into account the scanning time and the computation times as detailed in Table 4. We did not include the time duration of the information transfer to the computer. Nowadays, this additional time would still be negligible (a fraction of a second) in comparison with the duration shown in Table 4 and therefore preserve the expected added value of our smart scanning protocol. The next step of our investigation will be to implement this protocol on a real SEM-Raman microscope.

One may wonder why use the slow modality, Raman here, if the fast modality, SEM here, provides correlated, i.e. similar information. SEM provides similar information about the spatial structure of the sample while Raman spectroscopy enables characterization of the individual component based on molecular information. Concerning the specific practical use-case of bones, SEM can only address the degree of mineralization of the bone matrix, but certain bone pathologies are linked to abnormalities of the organic phase (osteolathyrism, Ehlers-Danlos, etc.) with a normal degree of mineralization. Raman provides access to both the mineral phase, with slightly more detailed information on the characterization of the mineral phase than SEM (degree of mineralization, carbonate content in the bone mineral, which impacts hardness and mechanical deformation of the mineral, mineral crystallinity), and the organic phase, with information on collagen and proteoglycans. Therefore, Raman is ideal for diagnosing bone pathology and the coupling of SEM and Raman for fast scanning is indeed an added value.

Beyond this proof of feasibility on a clinical sample for which time is a real limitation, one may wonder whether the proposed smart scanning protocol could be extended to other pairs of imaging modality. Specifically, one could think of other fast full-field imaging modalities which provide physical information such as polarization imaging for birefrengence contrasts, grazing lighting for surfacic patterns, or chemical information with standard color imaging. For each specific sample, it would then be necessary to demonstrate the correlation of the contrast carried by these modalities and the Raman microscopy.

The smart scanning of [22] can potentially be applied to any spectral imaging. It is expected to be of interest wherever the raster scan is considered as slow (as here for the bones samples in Raman). For instance, it was recently extended with success to Brillouin spectral imaging [31]. Here, also investigation on a dual-modality could be considered. For Brillouin, microscopy one could think of coupling with fluorescence microscopy or differential interference contrast as the fast full-field imaging modalities as these imaging modalities are correlated with the Brillouin information.

## 5. Conclusion

We have introduced an innovative smart-scanning protocol based on the use of two correlated modalities, here chosen as SEM and Raman for the considered illustrative example of bone sample analysis. It corresponds to an improvement of the recent Two-Pass protocol described in [22]. Tested here on bone samples, it has shown a reduction of the acquisition of a factor of about 100 compared to the high SNR Reference acquisition and by a factor of about 3 compared to the Two-Pass protocol. The focus on essential information also improved the statistical distinction between healthy and diabetic samples, hence accelerating the diagnosis process.

It is important to underline that the proposed approach is clearly distinct from the conventional correlative microscopy (SEM–Fluorescence, FIB–SEM,…) or the multi-modal standards in clinics (TEP–X-Ray, MRI–X-Ray,…) where the modalities are chosen to provide complementary, i.e. different information and the acquisition are performed jointly without exchange of information during the scan, while image registration of the imaging modalities is done in post-processing. With our multi-pass scanning approach, registration of the acquisition referential has to be done before hand, the modalities should be correlated and the information extracted from the fast modality is used to guide the scan of the slow modality. There is a potential for other dual modalities smart scanning protocols. However, the versatility of the method yet need to be investigated for other samples of biomedical interest.

## Data Availability

Data underlying the results presented in this paper are not publicly available at this time but may be obtained from the authors upon reasonable request.
